# Both radical prostatectomy following treatment with neoadjuvant LHRH agonist and estramustine and radiotherapy following treatment with neoadjuvant hormonal therapy achieved favorable oncological outcome in high-risk prostate cancer: a propensity-score matching analysis

**DOI:** 10.1186/1477-7819-12-134

**Published:** 2014-04-30

**Authors:** Takuya Koie, Chikara Ohyama, Hayato Yamamoto, Atsushi Imai, Shingo Hatakeyama, Takahiro Yoneyama, Yasuhiro Hashimoto, Tohru Yoneyama, Yuki Tobisawa, Masahiko Aoki, Yoshihiro Takai

**Affiliations:** 1 Department of Urology, Hirosaki University Graduate School of Medicine, 5 Zaifucho, Hirosaki 036-8562, Japan; 2 Department of Radiology, Hirosaki University Graduate School of Medicine, 5 Zaifucho, Hirosaki 036-8562, Japan

**Keywords:** Biochemical outcomes, Propensity-score matching, Prostate cancer, Radical prostatectomy, Radiation therapy

## Abstract

**Background:**

To date, the different treatment modalities for high-risk prostate cancer (Pca) have not been compared in any sufficiently large-scale, prospective, randomized clinical trial. We used propensity-score matching analysis to compare the oncological outcomes of high-risk prostate cancer between patients treated with radical prostatectomy (RP) and those treated with radiation therapy (RT).

**Methods:**

We studied 216 patients who received neoadjuvant therapy followed by RP (RP cohort) and 81 patients who received neoadjuvant androgen-deprivation therapy (ADT) followed by RT (RT cohort). The RP cohort received a luteinizing hormone-releasing hormone agonist and estramustine phosphate (280 mg/day) for 6 months prior to RP. The RT cohort received ADT for at least 6 months prior to RT using a 3-dimensional conformal radiotherapy technique. The total radiation dose was 70 to 76 Gy administered at 2 Gy/fraction.

**Results:**

Propensity-score matching identified 78 matched pairs of patients. The 3-year overall survival rates were 98.3% and 92.1% in the RP and RT groups, respectively (*P* = 0.156). The 3-year biochemical recurrence-free survival rates were 86.4% and 89.4% in the RP and RT groups, respectively (*P* = 0.878).

**Conclusions:**

Our study findings may suggest almost identical cancer control of RP and RT with appropriate neoadjuvant therapy in high-risk Pca. Therefore, issues of health-related quality of life may have an important impact on decision making in treatment of high-risk Pca.

## Background

Individuals with prostate-specific antigen (PSA) levels of ≥20 ng/mL, Gleason scores of ≥8, or clinical stage T2c/T3 tumors are defined as high-risk prostate cancer (Pca) patients [1]. Treatment options for high-risk Pca include external beam-radiation therapy (EBRT) with androgen-deprivation therapy (ADT); trimodal therapy with a combination of brachytherapy, EBRT, and ADT; and radical prostatectomy (RP) with neoadjuvant or adjuvant therapy. To date, no sufficiently large-scale, prospective, randomized clinical trials have compared the abovementioned treatment options. Thus, optimal management strategies for high-risk Pca patients have not been established. Previous studies comparing RP and EBRT were difficult to interpret because of biased treatment selection criteria, incomplete follow-up data, varied treatment protocols, and reliance on surrogate endpoints [2]. Above all, several studies comparing Pca treatment options have either overlooked medical comorbidities, because of lack of relevant information [3], or have attempted to control for measured comorbidities using statistical methods [4,5].

Therefore, we aimed to evaluate the overall survival (OS) and the biochemical recurrence-free survival (BRFS) rates of high-risk Pca patients who underwent either RP or EBRT using propensity-score matching analyses to adjust for treatment selection bias.

## Methods

### Patient selection

We conducted a retrospective chart review of 329 consecutive high-risk Pca patients treated at our institution between July 2004 and July 2012. Thirty-two patients who underwent only RP were excluded. We selected 216 patients who received neoadjuvant therapy followed by RP (the RP cohort) and 81 patients who received neoadjuvant ADT followed by EBRT (the RT cohort). The study protocol and informed consent documents was reviewed and approved by the Hirosaki University institutional review board.

### Treatment

A single pathologist reviewed the diagnostic biopsy specimens and surgical specimens. We have previously reported the active effect of luteinizing hormone-releasing hormone (LHRH) plus low-dose estramustine phosphate (EMP; LHRH + EMP) for high-risk Pca patients [6]. Patients in the RP cohort received LHRH (leuprolide (11.25 mg) or goserelin acetate (10.8 mg) every 3 months) and EMP (280 mg/day) for 6 months prior to RP [6]. Retropubic RP was performed as previously described in detail [7]. All patients in the RP cohort underwent the same lymphadenectomy procedure, which included removal of the bilateral obturator lymph nodes.

RT patients received ADT (LHRH and an antiandrogen) for at least 6 months prior to receiving EBRT. All patients were treated using a 3-dimensional conformal radiotherapy (3D-CRT) technique. The clinical target volume included the entire prostate and the bases of the seminal vesicles. A safety margin of 10 mm was added in all directions except posteriorly, where a 6-mm margin was added, to create the planning target volume. The total radiation dose was 70 to 76 Gy delivered in 2 Gy/fraction at 5 fractions/week.

### Follow-up evaluations

All patients were followed up by assessing serum PSA and testosterone levels every 3 months for 5 years and every 6 months thereafter. Pretreatment serum PSA levels were measured within 1 month of RP or EBRT administration. No patient was lost to follow-up in this study.

For RP-treated patients, disease recurrence or PSA failure was defined as serum PSA levels exceeding 0.2 ng/mL. If PSA levels did not decrease to less than 0.2 ng/mL after surgery, the date of RP was defined as the date of disease recurrence. For EBRT-treated patients, PSA failure was defined according to the 2006 consensus statement by the American Society of Therapeutic Radiation and Oncology [8]. PSA levels rising by 2 ng/mL or more above the nadir PSA levels is currently defined as biochemical failure after EBRT [8].

### Statistical analysis

To reduce the effect of treatment selection bias and potential confounding factors, we performed propensity-score matching analysis [9]. Propensity scores were calculated for each patient using multivariate logistic regression analysis including the following covariates: age, pretreatment PSA levels, biopsy Gleason scores, and clinical tumor staging. Tumors were staged according to the 2002 Staging Manual by the American Joint Committee on Cancer Staging [10]. The Gleason scores for prostate biopsy cores and surgical specimens were determined according to the 2005 guidelines by the International Society of Urological Pathology [11]. The predicted values according to the regression model estimated the propensity of each patient for receiving RP or EBRT according to his/her baseline characteristics. The differences between the two groups were assessed by fitting a logistic regression model using treatment as the response variable and baseline characteristics as covariables. Data were analyzed using IBM SPSS Statistics 20 software (International Business Machines Corp., New York, USA). OS and BRFS rates were analyzed using the Kaplan- Meier estimator. The relationship between survival and subgroup classification was analyzed using the log- rank test. All *P* values were two-sided and the significance level was set at *P* <0.05.

## Results

### Patient characteristics

Propensity-score matching identified 78 matched pairs of patients. Table 1 shows the pretreatment clinical characteristics of the two groups. No differences were noted in age, initial PSA levels, biopsy Gleason scores, or clinical T staging between the two groups. The median follow-up periods of the RP- and EBRT-treated patients were not significantly different.

**Table 1:** Pretreatment clinical characteristics categorized according to treatment administered to 156 patients with high-risk prostate cancer, adjusted for propensity scores

**Pretreatment characteristics**	**Radiation therapy (N = 78)**	**Radical prostatectomy (N = 78)**	***P***
Age (year, median)	73.5	71	0.0633^a^
Initial prostate-specific antigen level (ng/mL, median)	21.42	20.00	0.3886
Clinical T stage			
T1c	19	22	0.8482
T2	23	19	
T3	36	37	
Biopsy Gleason score			
≤6	5	4	0.9384
7	24	23	
≥8	49	51	
Follow-up period (month, median)	37.6	31.5	0.3338

^a^*P* values indicate statistical significance.

In the RT cohort, the median duration of ADT prior to receiving RT was 12 months (interquartile range: 9 to 16 months). Fifty-four patients (69%) received RT at a dose of 70 Gy, and 24 patients (31%) received RT at a dose of 74 Gy.

### Pathological outcomes in the radical prostatectomy cohort

All patients in the RP cohort were evaluated for pathological response. Regarding the pathological T stage, 5%, 59%, and 36% of patients had pT0, pT2, and pT3 tumors, respectively. Seven patients (9%) had positive surgical margins in the surgical specimens. None of the patients had received adjuvant therapy, including ADT or RT.

### Oncological outcomes

**Figure 1:**
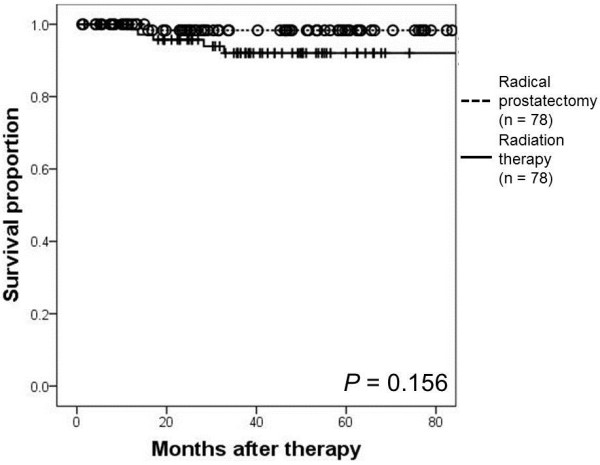
**Kaplan- Meier estimate of overall survival (OS).** The 3-year OS rate was 98.3% in the high-risk prostate cancer patients treated with neoadjuvant luteinizing hormone-releasing hormone agonist and estramustine phosphate followed by radical prostatectomy. The 3-year OS rate was 92.1% in patients treated with neoadjuvant androgen-deprivation therapy followed by radiation therapy (*P* = 0.156).

**Figure 2:**
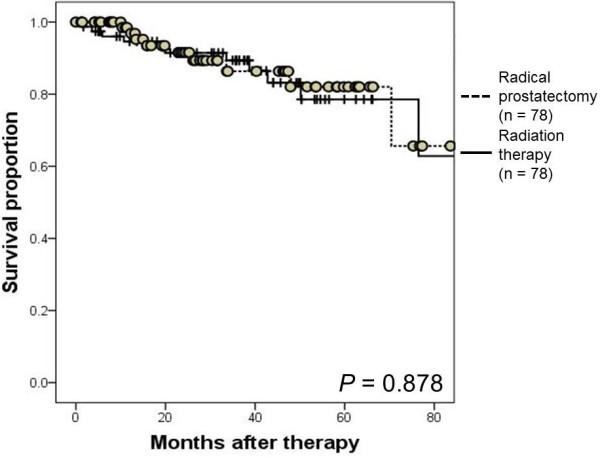
**Kaplan- Meier estimate of biochemical recurrence-free survival (BRFS).** The 3-year BRFS rates were 86.4% in the high-risk prostate cancer patients treated with neoadjuvant luteinizing hormone-releasing hormone agonist and estramustine phosphate followed by radical prostatectomy and 89.4% in those treated with neoadjuvant androgen-deprivation therapy followed by radiation therapy (*P* = 0.878).

The 3-year OS rates were 98.3% and 92.1% for the RP and RT cohorts, respectively (P = 0.156; Figure 1). At the time of analysis, 5 RT patients had died. The causes of death were prostate cancer, colorectal cancer, hepatocellular carcinoma, cerebral hemorrhage, and chronic heart failure. One patient from the RP cohort committed suicide. The 3-year BRFS rates were 86.4% and 89.4% in the RP and RT cohorts, respectively (P = 0.878; Figure 2). At the time of analysis, PSA failure had occurred in 11 RT and 9 RP patients. These patients did not show clinical recurrence except for 1 RT-treated patient. In the RP cohort, the 3-year BRFS rate was 89.9% (95% confidence interval (CI): 66.5 to 77.7) in patients who achieved pathological T0/T2 status, and 78.1% (95% CI: 45.4 to 73.9) in those with T3 status (P = 0.018).

## Discussion

To our knowledge, the efficacies of EBRT and RP in terms of biochemical outcomes, particularly in high-risk Pca patients, have not been compared in randomized controlled trials; therefore, reliance on observational data has become mandatory. A comparative analysis of studies involving prostate cancer treated with various modalities was conducted by the Prostate Cancer Results Study Group. The analysis, authored by Grimm *et al*., provides some insight into the relative effectiveness of surgery and RT for high-risk disease [12]. Combination therapies involving RT and brachytherapy plus or minus ADT appear superior to more localized treatments such as RP alone or RT alone [12]. In the present study, OS and PSA outcomes following RP or 3D-CRT were compared among high-risk Pca patients who were matched for pretreatment predictors.

We administered neoadjuvant LHRH + EMP followed by RP to high-risk Pca patients. Among high-risk Pca patients, reported rates of PSA-free survival after RP alone was 35 to 62% [13,14]. Furthermore, neoadjuvant hormone therapy before RP reduces the rate of positive surgical margins, potentially resulting in pathologic complete responses. However, neoadjuvant ADT has not been shown to be beneficial for patient outcomes, especially in terms of PSA-free survival, in randomized trials [15]. Long-term administration of low-dose EMP may have a positive impact on the PSA-free survival rate; the PSA-free survival rate was 86.4% in our study, which was higher than the values reported by several other clinical trial [13-15].

In EBRT-treated high-risk Pca patients, an RT dose of 70 Gy may be inadequate to eradicate the disease completely. Support for this hypothesis came from a randomized dose escalation trial (78 Gy versus 70 Gy) [16], in which a beneficial effect was noted for all patients in terms of the 5-year BRFS rate (78% versus 68%; *P* = 0.03), and particularly, in patients with a pretreatment PSA level of >10 to 20 ng/mL (72% versus 43%; *P* = 0.01). Patients with locally advanced Pca were found to experience favorable survival outcomes in a prospective randomized clinical trial when ADT was added to EBRT [17].

In fact, a more favorable outcome may be achieved with neoadjuvant LHRH + EMP and RP or 3D-CRT and ADT than RP or 3D-CRT alone in high-risk Pca patients. Our study findings may suggest almost identical cancer control by RP and RT with appropriate neoadjuvant therapy in high-risk Pca. On the other hand, it is interesting to note that benefits in terms of BRFS were not observed after treatment completion in the two groups in the present study. This observation may be attributable to the differing definition of PSA failure between RP- and EBRT-treated patients. PSA must reach the nadir value in patients treated with EBRT, and this can take 1 to 2 years or occasionally longer [18]. Conversely, almost all RP-treated patients will achieve the nadir PSA value within 1 to 2 months or sooner after therapy. Therefore, clinically meaningful and reliable results require longer follow-up periods.

Finally, the current study was not performed as a non-inferiority study to compare the efficacy of RP with the RT in patients with high-risk Pca. Our findings were limited by the retrospective nature of our study and the relatively small study sample size. The RP patients received neoadjuvant LHRH + EMP, and RT patients received neoadjuvant LHRH and antiandrogen. Propensity-score analysis is a method used to reduce bias in observational studies and matching was limited to available variables. Additionally, other factors such as quality of life, continence, and erectile function, which also affect treatment decisions, were not evaluated in our study. Therefore, issues of health-related quality of life may have an important impact on decision making of treatment in high-risk prostate cancer. However, our study results may assist in decision-making for managing high-risk Pca patients because prospective randomized clinical trial data are lacking. Future clinical trials are warranted.

## Conclusions

Our study findings may suggest almost identical cancer control of RP and RT with appropriate neoadjuvant therapy in high-risk Pca. Therefore, issues of health-related quality of life may have an important impact on decision making in treatment of high-risk Pca.

## Abbreviations

ADT: androgen-deprivation therapy; BRFS: biochemical recurrence-free survival; EBRT: external beam-radiation therapy; LHRH + EMP: luteinizing hormone-releasing hormone agonist and estramustine phosphate; OS: overall survival; Pca: prostate cancer; PSA: prostate-specific antigen; RP: radical prostatectomy; RT: radiation therapy; 3D-CRT: 3-dimensional conformal radiotherapy.

## Competing interests

The authors declare that they have no competing interests.

## Authors’ contributions

TK wrote the manuscript. HY, AI, SH, TY and MA performed clinical follow-up examinations and contributed to the manuscript. YH reviewed the pathological specimens. YT, TY and YT contributed to manuscript drafting. CO was responsible for the concept, design, data interpretation, and critical revision of the manuscript. All authors read and approved the final version of the manuscript.
